# Regulatory Status of Genome-Edited Organisms Under the Japanese Cartagena Act

**DOI:** 10.3389/fbioe.2019.00387

**Published:** 2019-12-06

**Authors:** Mai Tsuda, Kazuo N. Watanabe, Ryo Ohsawa

**Affiliations:** ^1^Faculty of Life and Environmental Sciences, University of Tsukuba, Tsukuba, Japan; ^2^Tsukuba Plant Innovation Research Center (T-PIRC), University of Tsukuba, Tsukuba, Japan

**Keywords:** genome editing, regulatory status, Japan, Cartagena Protocol, LMOs

## Abstract

The Japanese government recognizes the substantial values of genome-edited agricultural organisms and has defined in which cases these are covered by the existing regulatory framework to handle this technology. Genome-editing technologies could revolutionize and accelerate plant breeding owing to the simplicity of the methods and precision of genome modifications. These technologies have spread rapidly and widely, and various genome-edited crops have been developed recently. The regulatory status of genome-edited end products is a subject of controversy worldwide. In February 2019, the Japanese government defined genome-edited end products derived by modifications of SDN-1 type (directed mutation without using a DNA sequence template) as not representing “living modified organisms” according to the Japanese Cartagena Act. Here, we describe the classification and regulatory status of genome-edited end products in this decision. We hope that reporting the progress in Japan toward the implementation of this regulatory approach will provide insight for scientific and regulatory communities worldwide.

## Introduction

Article 8 (g) of the Convention on Biological Diversity (CBD)^1^ establishes the obligation to Parties to “establish or maintain means to regulate, manage or control the risks associated with the use and release of living modified organisms (LMOs).” Building on that, Article 1 of the Cartagena Protocol on Biosafety (CPB) aims “to contribute to ensuring an adequate level of protection in the field of the safe transfer, handling and use of living modified organisms resulting from modern biotechnology that may have adverse effects on the conservation and sustainable use of biological diversity, taking also into account risks to human health, and specifically focusing on transboundary movements.”^2^ According to the general provisions in Article 2 of the Protocol,^2^ each party shall take necessary and appropriate legal, administrative, and other measures to implement its obligations under this Protocol. Because the Cartagena Protocol was established in awareness of future technological developments, it has to be considered to what extent it applies to organisms derived by genome-editing techniques.

Since the Eighth meeting of the Conference of the Parties serving as the meeting of the Parties to the Cartagena Protocol (COP/MOP-8) in 2016^3^, genome editing has become a major focus. At COP14, it was agreed that “broad and regular horizon scanning, monitoring and assessing of the most recent technological developments is needed [“taking into account that this may include genome editing”] for reviewing new information regarding the potential positive and potential negative impacts of synthetic biology *vis-à-vis* the three objectives of the Convention and those of the Cartagena Protocol and Nagoya Protocol”^4^.

Japan is a Party to the Cartagena Protocol. In 2003, the domestic “Act on the Conservation and Sustainable Use of Biological Diversity through Regulations on the Use of Living Modified Organisms” for implementing the Cartagena Protocol (called the Cartagena Act) was established^5^. The Japanese government has been proactively looking at the organisms of genome editing, and on February 8, 2019^6^, decided that some genome-edited organisms should be considered as LMOs while others are not subject to the Cartagena Act. No announcement of the publication of this notice has been made in any foreign language except for a short English flier by the Ministry of the Environment (MOE)^7^, so here we would like to analyze and explain its content for an international audience. In general, Japan has been rather slow in implementing the regulatory framework on biotechnology (Watanabe et al., 2004), and the present notification on genome editing may facilitate more timely development of commercial products. This interpretation of the regulatory framework in Japan could encourage other countries to consider similar balanced legislation.

## Current Regulatory Status Of Genome-Edited Organisms Worldwide

The Organization for Economic Co-operation and Development (OECD) Working Group for the Harmonization of Regulatory Oversight in Biotechnology has discussed the safety and regulatory considerations raised by genome-edited organisms. In June 2018, “The OECD Conference on Genome Editing: Applications in Agriculture—Implications for Health, Environment and Regulation” was held in Paris^8^.

Organisms developed through new breeding techniques, including genome editing, may contain nucleic acids from a foreign source. The regulatory status of genome-edited organisms has been discussed in various countries, and the regulatory approaches differ across countries. On March 28, 2018, the U.S. Secretary of Agriculture Sonny Perdue stated that the U.S. Department of Agriculture (USDA) “does not regulate or have any plans to regulate plants that could otherwise have been developed through traditional breeding techniques, as long as they are not plant pests or developed using plant pests”^9^. In Argentina (Whelan and Lema, 2015), Chile (Cameron et al., 2017), and Brazil (Chandrasekaran et al., 2016), the status of organisms obtained through new plant breeding techniques requires confirmation that they have no nucleic acids derived from foreign organisms.

On July 25, 2018, the Court of Justice of the European Union issued its judgment that “organisms obtained by means of techniques/methods of mutagenesis constitute GMOs within the meaning of that provision” and “only organisms obtained by means of techniques/methods of mutagenesis which have conventionally been used in a number of applications and have a long safety record are excluded from the scope of that directive” under the directive 2001/18/EC (Official Journal of the European Communities, 2001). This ruling has resulted in much uncertainty and discussion regarding the regulatory status of genome-edited organisms in general^10^.

Two countries in Oceania have different regulations. Australia gave notice of “Gene Technology Amendment (2019 Measures No. 1) Regulations 2019,” which is modified law of “The Gene Technology Act 2000” on April 4, 2019^11^. The Australian government will not regulate the use of gene-editing techniques in plants, animals, and human cell lines that do not introduce a novel combination of genetic material (Mallapaty, 2019). According to Fritsche et al. (2018), “in 2014, New Zealand's Environmental Protection Authority ruled that plants produced via genome-editing methods, where no foreign DNA remained in the edited plant, would not be regulated as LMOs,” but this decision was overturned by the High Court; currently, New Zealand considers all gene-edited organisms as LMOs.

## History Of Discussion On Regulatory Status Of Genome-Edited Organisms In Japan

Under the Cartagena Act, the use of living modified crops requires reviews of the environmental risk to biodiversity associated with the deliberate release of such crops. The Cartagena Act states that LMOs are regulated in terms of the final products as “living organisms having nucleic acids obtained by utilizing a technique for processing nucleic acids outside the cell for the purpose of transferring or replicating the nucleic acids by transferring them into a cell, virus, or viroid” (Chapter I, Article 2, item 2)^5^ in accordance with items (g) and (h) of the “Use of Terms” of Article 3 of the CPB^2^. An LMO is any organism with inserted extracellularly processed nucleic acid (including RNA)^7^. If the end products of genome-editing technology have no remnants of inserted nucleic acid or its replicated product and are undistinguishable from those developed *via* traditional breeding methods, they are not LMOs. In the Cartagena Act, a “replicated product” is replicated nucleic acid from transformed nucleic acid that is neither RNA nor protein.

In Japan, the regulatory perspective of genome-edited end products has been discussed over the past 5 years. In August 2014, the Science Council of Japan released the report “Current status and problems of new plant breeding technology (NPBT)”^12^. This report stated that knowledge accumulation and management operations according to the Cartagena Act are important for crop development using NPBT. In September 2015, the New Plant Breeding Technique Study Group, established at the Secretariat of Agriculture, Forestry and Fisheries Research Council, the Ministry of Agriculture, Forestry and Fisheries (MAFF), released the document “Toward the development and practical application of crops using new plant breeding techniques (NPBTs) such as genome editing”^13^, which asserted that appropriate measures will be implemented under the Cartagena Act for dealing with living organisms with foreign genes transiently introduced during breeding, and international harmonization on regulatory status will be promoted. In August 2016, at the Expert Committee on LMOs of the Nature Conservation Committee, the Central Environment Council, MOE issued a report entitled “Examining enforcement of the Cartagena Act,” which stated that decision making on regulatory status of organisms that do not contain exogenous nucleic acids created by new breeding techniques such as genome editing is an urgent issue, and it is necessary to carefully consider this status in light of the latest scientific knowledge and international harmonization^14^. In September 2016, a member of the House of Councilors submitted the document “Subjective Questionnaire on Genetic Research, Development, and Regulation of Genome Editing Technology” to the Cabinet Office^15^. The Council of Science, Technology and Innovation of the Cabinet Office has established the Working Group for Bio-Strategy, and the interim report of the Working Group was issued in June 2018^16^. This report suggested that clarification of the regulatory status of genome-edited crops under the Cartagena Act and Food Sanitation Act is at an early stage, and promotion of public understanding of genome-editing techniques is needed. Later in the same month, the Cabinet Office endorsed the Integrated Innovation Strategy^17^, which stated that the regulatory status of organisms obtained by genome editing in line with the Cartagena Act and the regulatory status of agricultural and fishery organisms obtained using this technology under the Food Sanitation Act should be clarified by the end of fiscal year 2018, and efforts should be made to promote international harmonization. On July 11, 2018, The Expert Meeting on Genome Editing Technologies under the Cartagena Act was established within the Expert Committee on LMOs of the Nature Conservation Committee, the Central Environment Council, MOE as the administration of the Cartagena Act^18^. On August 7, the meeting was held for the first time to discuss the regulatory status of genome-editing technology under the Cartagena Act^19^; on August 20, the second meeting summarized the discussion on the regulatory classification and status of genome-editing technology as a draft report^20^. On August 30, the 2nd Expert Committee on LMOs produced the draft report entitled “Classification and status of organisms produced by application of genome-editing technology under the Cartagena Act”^21^. In 2018 (September 20–October 19), a public consultation on the proposal was arranged^22^. On January 21, 2019, the feedback was discussed at the Nature Conservation Committee, the Central Environment Council^23^, and on February 8, 2019, the final decision was reported by the MOE^6^. Here, we report the key elements of the final decision made by the MOE.

## Regulatory Status Of Genome-Edited Organisms In Japan

**Figure 1:**
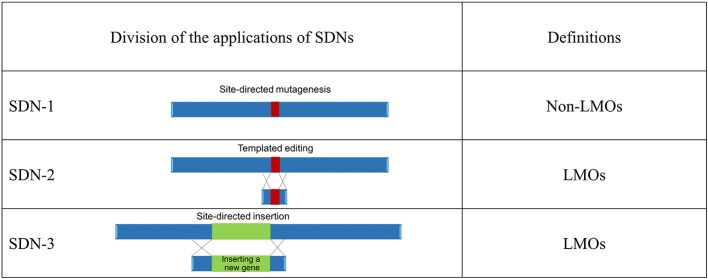
Regulatory overview of genome-edited organisms in Japan. Ministry of the Environment presented a preliminary draft^21^ to define organisms produced using three applications of site-directed nucleases (SDNs). Living modified organisms (LMOs): any living organism that possesses a novel combination of genetic material obtained through the use of modern biotechnology^2^.

Genome-editing techniques are classified into three principal categories, site-directed nuclease (SDN)-1, that is, site-directed mutagenesis, SDN-2, that is, templated editing, and SDN-3, that is, site-directed gene insertion (Figure 1). This categorization is based on the definition by Lusser et al. (2011, 2012). The types of artificial nucleases, which include zinc finger nucleases (ZFNs), transcription activator-like effector nucleases (TALENs), and clustered regularly interspaced short palindromic repeats (CRISPR), used for targeted modification (Podevin et al., 2013) are considered.

The end products from the SDN-1 methods do not contain inserted nucleic acid or its replicated product, so they do not satisfy the definition of LMOs in the Cartagena Act (Chapter I, Article 2, item 2)^5^. On the other hand, the end products obtained by the SDN-2 and SDN-3 methods contain inserted nucleic acids processed extracellularly and are categorized as LMOs. This categorization is the same as in a document issued by the Australian Government^24^. The size of the nucleic acid insert is undefined in the Cartagena Act. Any organism with inserted extracellularly processed nucleic acid (including RNA) is regarded as an LMO and is subject to the regulations stipulated in the Cartagena Act unless the complete removal of the inserted nucleic acid (including RNA) or its replicated product is confirmed. The final determination according to the MOE^6^ approach would be applicable to null segregants, in which the inserted foreign gene is segregated out through backcrossing.

In the future, the newly developed biotechnological end products have to be thoroughly classified in terms of whether or not they contain extracellularly processed nucleic acids. Technology users are requested to notify the government with information on unregulated end products created through genome-editing technology, including the details of their production and any knowledge of their impact on biodiversity^4^ prior to use. Competent national authorities [administrative agencies, such as the MAFF, the MOE, and the Ministry of Education, Culture, Sports, Science and Technology (MEXT)] call on users of genome-editing SDN-1-based technologies to submit a review of the biological characteristics and impact on biodiversity of genome-edited organisms to the appropriate ministry. Submission is not needed if there has been no change to a previously submitted review, or genome-edited organisms are used in an environment in which containment measures have been taken.

In the case of a probable risk to biodiversity, the competent national authority will require additional information from the user; then, necessary measures can be taken. MOE will post-annually some information on unregulated end products, mainly the taxonomical species of the modified organism, change of traits added by the modification, usage of the organism, and discussion on possible influences on biological diversity when the organism is used; all information to be provided is listed in a flyer in English produced by the MOE^7^, and the names of the administrative agency to notify depending on the use of the organism, on the website^6^,^7^. In case of any concern about the impact of a genome-edited organism on biodiversity, the user must take necessary measures to mitigate the effect on biodiversity immediately according to the Cartagena Act and promptly report this to the administrative agencies in charge, which would take appropriate measures in consideration of the public policy on biodiversity conservation. The administrative agencies can also require additional information upon considering the characteristics of the species.

## Toward Future Decision Making On Genome-Edited Organisms In Japan

At first, in response to the draft by the MOE^21^, the Japanese Society of Breeding made a statement on October 1, 2018^25^. The Society appreciated that users are requested to provide information on genome-edited organisms that are not subject to the Cartagena Act. The Society stated that if this proposed policy enters Japanese legislation, breeding institutions, universities, and seed companies can make substantial contributions to the stable supply of food through the improvement of plants using genome-editing technology. The administrative agencies such as MAFF, MOE, MEXT, and the Ministry of Health, Labor, and Welfare should work together to clarify procedures for providing information for the use of genome-edited organisms. These regulations will promote practical use of superior crop varieties generated through genome-editing technology.

Although the latest Japanese government regulation was noticed on February 8, 2019^6^, the scientific aspects, such as the method for assessing the persistence of a foreign gene region in a null segregant and the effects of unintentional mutations including off-target effects, need to be clarified. A method has been established for confirming the persistence of a foreign DNA fragment by using a next-generation sequencer and improved Southern hybridization, which is outlined in Tabei (2019). The current methods for detection of DNA sequence alterations through genome-editing techniques were summarized in the European Network of GMO Laboratories^26^, and appropriate judgment criteria and detection methods are being discussed worldwide.

## Conclusions And Recommendations

Japan has decided on rules for regulatory status of genome-edited organisms^6^. The organisms produced by SDN-1 are not subject to regulation under the Cartagena Act, as they are considered similar to those produced by conventional breeding technologies. Although mutations in the organisms produced by SDN-2 are equivalent to those that occur naturally, such organisms are considered LMOs under the Cartagena Act if they possess inserted extracellularly processed nucleic acid. The regulatory status of organisms produced by SDN-2 is considered on a case-by-case basis worldwide. Organisms produced by SDN-3 are considered LMOs. The decision of the MOE of Japan makes it possible for each stakeholder to judge the actions needed on the basis of defined criteria. We hope that the availability of this information will promote the use of genome editing for plant breeding under the proper regulatory status of the Cartagena Act in Japan and will be helpful for future discussions at the OECD and regulatory decision making in other countries.

## Author Contributions

MT organized the manuscript. KW provided input on international legal instruments. RO verified the information on domestic regimes. All authors discussed the results and contributed to the final manuscript.

### Conflict of Interest

The authors declare that the research was conducted in the absence of any commercial or financial relationships that could be construed as a potential conflict of interest.

## Footnotes

^1^Convention on Biological Diversity (CBD). *Article 8, in situ Conservation*. Available online at: https://www.cbd.int/convention/articles/default.shtml?a=cbd-08 (accessed October 8, 2019).

^2^CBD. *Text of the Cartagena Protocol on Biosafety*. Available online at: https://bch.cbd.int/protocol/text (accessed October 8, 2019).

^3^CBD. *Outline of Guidance on Risk Assessment Of Living Modified Organisms Developed Through Synthetic Biology, UNEP/CBD/BS/COP-MOP/8/8/ADD3*. Available online at: https://www.cbd.int/doc/meetings/bs/mop-08/official/bs-mop-08-08-add3-en.pdf (accessed October 8, 2019).

^4^CBD. *Synthetic Biology, Draft Decision Submitted by the Chair of Working Group II, CBD/COP/14/WG.2/CRP.20*. Available online at: https://www.cbd.int/doc/c/043c/a200/78251e44a6f7ceed13b44312/cop-14-wg-02-crp-20-en.pdf (accessed October 8, 2019).

^5^Japan Biosafety Clearing House (J-BCH). *Domestic Law and Regulations*. Available online at: https://www.biodic.go.jp/bch/english/law.html (accessed October 8, 2019).

^6^Ministry of the Environment (MOE). *About the Handling of Organisms Produced by the Use of Genome Editing Technology that Do Not Match the Definition of “Genetically Modified Organisms” in the Cartagena Act*. Available online at: http://www.biodic.go.jp/bch/download/genome/genome_tsuuchi20190208.pdf (accessed June 14, 2019).

^7^MOE. *To Genome Editing Technologies Users*. Available online at: https://www.env.go.jp/press/2_2_%20genome%20editing_En.pdf (accessed July 18, 2019).

^8^OECD. *The OECD Genome Editing Hub, OECD Conference on Genome Editing: Applications in Agriculture on 28–29 June 2018*. Available online at: http://www.oecd.org/environment/genome-editing-agriculture/ (accessed June 14, 2019).

^9^U.S. Department of Agriculture (2018). *Secretary Perdue Issues USDA Statement on Plant Breeding Innovation*. Washington, DC. Available online at: https://www.usda.gov/media/press-releases/2018/03/28/secretary-perdue-issues-usda-statement-plant-breeding-innovation (accessed June 14, 2019).

^10^Court of Justice of the European Union. *Judgement of the Court in Case C-528/16: Court of Justice of the European Union*. Available online at: http://curia.europa.eu/juris/document/document.jsf?text=&docid=204387&pageIndex=0&doclang=EN&mode=lst&dir=&occ=first&part=1&cid=138460 (accessed October 8, 2019).

^11^Federal Register of Legislation. *Gene Technology Amendment (2019 Measures No. 1) Regulations 2019*. Available online at: https://www.legislation.gov.au/Details/F2019L00573 (accessed July 31, 2019).

^12^Science Council of Japan. *Current Status and Problems of New Plant Breeding Technology (NPBT)*. Available online at: http://www.scj.go.jp/ja/info/kohyo/pdf/kohyo-22-h140826.pdf (accessed June 14, 2019).

^13^New Plant Breeding Technique Study Group (2015). *Toward the Development and Practical Application of Crops Using New Plant Breeding Techniques (NPBTs) Such as Genome Editing*. Available online at: http://www.affrc.maff.go.jp/docs/commitee/nbt/attach/pdf/top-2.pdf (accessed June 14, 2019).

^14^MOE. *Minute of the Expert Committee on LMOs (Third in Fiscal Year 2016)*. Available online at: https://www.env.go.jp/council/12nature/y127-03a.html (accessed June 14, 2019).

^15^House of Councilors, The National Diet of Japan. *Subjective Questionnaire on Genetic Research, Development, and Regulation of Genome Editing Technology of the 192nd Diet*. Available online at: http://www.sangiin.go.jp/japanese/joho1/kousei/syuisyo/192/meisai/m192004.htm (accessed June 14, 2019).

^16^Cabinet Office. *Working Group for Bio-Strategy and the Interim Report*. Available online at: https://www8.cao.go.jp/cstp/tyousakai/bio/bio_chukan.pdf (accessed June 14, 2019).

^17^Cabinet Office. *The Integrated Innovation Strategy*. Available online at: http://www8.cao.go.jp/cstp/senryakukaigi/3kai/siryo3.pdf (accessed June 14, 2019).

^18^MOE. *Minute of the Expert Committee on LMOs (First in Fiscal Year 2018)*. Available online at: https://www.env.go.jp/council/12nature/02_3.html (accessed June 14, 2019).

^19^MOE. *Minute of the Expert Meeting on Genome Editing Technologies under the Cartagena Act (First in Fiscal Year 2018)*. Available online at: https://www.env.go.jp/council/12nature/post_56.html (accessed June 14, 2019).

^20^MOE. *Minute of the Expert Meeting on Genome Editing Technologies under the Cartagena Act (Second in Fiscal Year 2018)*. Available online at: https://www.env.go.jp/council/12nature/30_3.html (accessed June 14, 2019).

^21^MOE. *Minute of the Expert Committee on LMOs (Second in Fiscal Year 2018)*. Available online at: https://www.env.go.jp/council/12nature/30_10.html (accessed July 31, 2019).

^22^MOE (2018). *Call for Public Comments on “Classification and Handling of Organisms Produced by Application of Genome Editing Technology under the Cartagena Act.”* Available online at: https://www.env.go.jp/press/105960.html (accessed June 14, 2019).

^23^MOE (2019). *The Nature Conservation Committee, the Central Environment Council. (37th)*. Available online at: https://www.env.go.jp/council/12nature/37_3.html (accessed July 31, 2019).

^24^Office of the Gene Technology Regulator. *Technical Review of the Gene Technology Regulations 2001-2016 Discussion Paper Consultation*. Available online at: http://www.ogtr.gov.au/internet/ogtr/publishing.nsf/Content/reviewdiscussionpaper-htm (accessed July 18, 2019).

^26^European Network of GMO Laboratories (ENGL) (2019). *Detection of Food and Feed Plant Products Obtained by New Mutagenesis Techniques (JRC116289)*. Available online at: http://gmo-crl.jrc.ec.europa.eu/doc/JRC116289-GE-report-ENGL.pdf (accessed October 8, 2019).
